# Performance Evolution and Formulation Improvement of Resin-Based Anchoring Materials for Hydrochemical Environments

**DOI:** 10.3390/ma18204741

**Published:** 2025-10-16

**Authors:** Wenhui Bian, Meiqiang Dong, Kexue Wang, Zhicheng Sun, Ziniu Wang, Shuyi Zhao, Jun Yang

**Affiliations:** 1 Department of Hydraulic Engineering, Tsinghua University, Beijing 100084, China; bianwh@mail.tsinghua.edu.cn; 2 State Key Laboratory for Tunnel Engineering, China University of Mining and Technology, Beijing 100083, China; yangjun_cumtb@163.com; 3 Beijing Digit Rock Technology Co., Ltd., Beijing 100083, China; 4 School of Mechanics and Civil Engineering, China University of Mining and Technology, Beijing 100083, China; 19975509007@163.com (M.D.); sunzhic01124@163.com (Z.S.); wzn512@163.com (Z.W.); 19159096713@163.com (S.Z.)

**Keywords:** water-bearing rock mass, resin anchoring agent, pull-out behavior, formulation improvement, ring shear test

## Abstract

The performance of resin anchoring agents in deep coal mine roadways is significantly compromised by water-bearing and chemically aggressive conditions, posing a major threat to support system reliability. This study aims to systematically quantify this performance deterioration and develop a more resilient material solution for these challenging environments. A comprehensive experimental program was conducted, including uniaxial compression, pull-out, and interface shear tests, accompanied by the systematic improvement of the resin formulation and microstructural analysis via Scanning Electron Microscopy (SEM). The results showed that increasing borehole water content to 30% reduced the compressive strength of conventional resin by over 40%, while acidic environments (pH = 5) caused a 70% drop in its interfacial shear strength. In contrast, an improved formulation incorporating hydroxypropyl acrylate and a super absorbent polymer (SAP) exhibited a 20% higher initial strength, maintained over 85% of its strength under water saturation, and retained functional residual strength in acidic conditions. SEM analysis confirmed that the improved resin’s denser microstructure suppressed interfacial microcrack formation. The findings demonstrate that the improved formulation provides a robust material basis for enhancing the long-term durability and safety of anchorage support systems in extreme underground engineering environments.

## 1. Introduction

With the continuous increase in coal mining depth in China, the hydrogeological conditions encountered in deep coal mine roadways have become increasingly complex, posing severe challenges to the reliability and durability of surrounding rock support technologies [1,2,3]. Deep rock masses often exhibit pronounced discontinuities and fragmentation, and the development of fracture networks significantly enhances interactions between groundwater and surrounding rock, leading to the deterioration of mechanical properties and a reduction in the stability of anchorage systems, thereby threatening roadway safety [4,5]. As a core bonding material for bolted support systems, the long-term durability and stability of resin anchoring agents under complex water-bearing conditions directly determine the effectiveness and safety of the entire support structure. While various grouting materials like self-compacting concrete exist, resin-based anchoring agents are often preferred in deep coal mine roadways due to their rapid curing speed, high early strength, and excellent chemical resistance, which are critical for ensuring immediate support in dynamic and often corrosive underground environments [6]. Both engineering practice and laboratory studies have demonstrated that water can significantly impair the bonding strength and curing efficiency of resin anchoring agents, leading to anchorage failure and even engineering accidents [7,8].

Existing studies have attributed this performance degradation primarily to the hindrance of polymerization reactions and the reduction in interfacial bonding strength under water-bearing conditions. Kang et al. [9], through pull-out tests, found that increasing borehole moisture content markedly reduces the shear strength at the resin–rock interface and alters the failure mode. Wang et al. [10] reported that when water content exceeds a critical threshold, the anchorage bearing capacity drops sharply by more than 50%. Recent studies have further shown that moisture ingress can plasticize the polymer matrix, reducing its glass transition temperature and stiffness, which compromises load transfer at the interface [11]. Numerical simulations based on finite element and discrete element methods further revealed that the presence of water leads to uneven shear stress distribution at the interface and accelerates crack initiation and propagation, thus undermining the long-term reliability of the anchorage system [12]. Zhao et al. [13] demonstrated that increasing anchorage length can partially improve interface shear strength under water-bearing conditions, but this strategy fails to fundamentally resolve long-term performance degradation issues.

In addition to water, acidic and alkaline groundwater environments further exacerbate interfacial degradation of resin anchoring agents. Ajir [14], combining mechanical testing with scanning electron microscopy (SEM), found that acid–base environments significantly reduce interfacial bonding capacity and severely damage the resin’s microstructure. The primary corrosion mechanism involves hydrolysis of ester bonds in the resin matrix, where acidic conditions promote bond cleavage via hydrogen ion attack, while alkaline conditions degrade the polymer via saponification reactions [15,16]. This chemical aging is particularly detrimental to the long-term creep and fatigue resistance of the anchorage system [17]. He [18] and Zhong et al. [19], respectively, confirmed that both shear and compressive strengths of resin anchoring agents decline significantly with prolonged exposure to corrosive environments, increasing the risk of premature interface failure.

Despite these valuable insights, the existing body of literature reveals several critical research gaps. First, most studies focus on the influence of a single factor (either water or a specific chemical solution) in short-term tests. There is a significant lack of comprehensive research on the performance evolution of resin anchors under the more realistic, coupled effects of hydro-chemical environments, where moisture and aggressive ions act simultaneously [20]. Second, the majority of research has concentrated on characterizing the problem of performance loss in traditional unsaturated polyester resins. There is a pressing need for a systematic approach to developing and validating improved, high-performance formulations specifically designed to counteract these known failure modes [21,22]. While polymer-modified materials such as hydroxypropyl acrylate resins and super absorbent polymers (SAP) have shown promise in related fields [23,24,25], their synergistic effect and optimization within a complete anchoring system for these coupled conditions remain largely unexplored [26,27].

While specific ultimate force requirements for rock bolts can vary significantly depending on the geological conditions, support design, and local regulations, a common benchmark for roadway support in coal mines is to maintain a long-term anchorage capacity well above 100 kN [28]. However, the primary aim of this study is not to meet a specific force threshold, but rather to conduct a comparative analysis. The main task is to systematically quantify the relative performance deterioration of a conventional anchor in adverse environments and to demonstrate the efficacy of an improved formulation in mitigating these effects. The performance under standard dry conditions serves as a benchmark for this comparative evaluation.

Therefore, this study aims to address these research gaps. Building on recent advancements in polymer modification, this study provides a holistic understanding of resin anchor performance deterioration and enhancement. Specifically, we first quantitatively evaluate the performance of a conventional resin anchor system under varying water contents and anchorage lengths. Based on these baseline findings, we then develop and systematically improve a high-performance resin formulation by incorporating hydroxypropyl acrylate resin and Super Absorbent Polymer (SAP) to enhance water resistance and mechanical strength. Furthermore, the study investigates the interfacial shear behavior and microstructural degradation mechanisms of both conventional and improved resins under simulated acidic and alkaline groundwater conditions. Ultimately, this multi-faceted approach aims to provide a robust material design basis and practical solutions for anchorage support in deep, chemically aggressive, and water-bearing rock masses.

## 2. Experimental Investigation on the Anchoring Performance Under Water-Bearing Conditions

Deep coal mine roadways are frequently subjected to dripping or gushing water, and such water-bearing environments significantly affect the setting behavior and performance stability of resin anchoring agents, thereby compromising the reliability of the support system and potentially leading to failure [29]. To systematically investigate the specific influence of moisture on anchorage performance, this study conducted pull-out tests under varying moisture contents and anchorage lengths, and further employed uniaxial compression tests to explore the mechanisms by which water content influences the strength characteristics of the anchoring material.

### 2.1. Experimental Materials and Specimen Preparation

**Figure 1:**
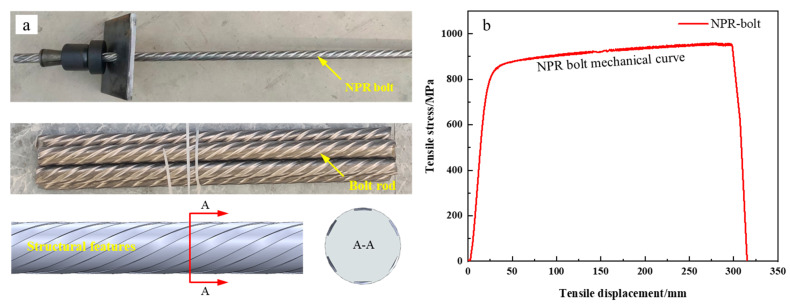
The NPR bolt. (**a**) Photograph showing the physical structure. (**b**) Typical load–displacement curve under tension.

**Figure 2:**
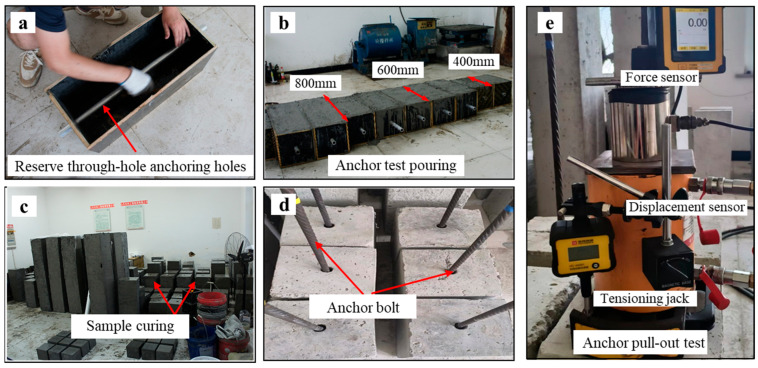
Preparation process of anchorage specimens: (**a**) Prefabricated mold; (**b**) Casting of specimens with different anchorage lengths; (**c**) Curing; (**d**) Completion of anchorage; (**e**) Tensioning operation.

The pull-out tests utilized Negative Poisson’s Ratio (NPR) bolts with a diameter of 18 mm and a yield strength of 850 MPa [30,31]. This type of bolt features high prestress and constant resistance characteristics, thus imposing more stringent requirements on anchoring quality. The bolt’s physical appearance and its typical load–displacement behavior are illustrated in Figure 1. The surrounding rock was simulated using C25-grade concrete, mixed at a ratio of water: cement: sand: gravel = 0.58:1:1.2:2.32, with specimen dimensions of 250 mm × 250 mm. Custom molds were used to cast rectangular specimens (Figure 2a), in which 30 mm-diameter aluminum alloy tubes were embedded to simulate boreholes for anchorage.

To cover the typical anchorage length range used for resin bolts, three lengths—400 mm, 600 mm, and 800 mm—were designed (Figure 2b). The concrete specimens were cured under standard conditions for 30 days to ensure strength stability (Figure 2c). The anchoring agent selected was a medium-speed curing resin (MSZ2360), with a gelation time ranging from 90 to 180 s, ensuring sufficient mechanical stability for the anchorage system. A typical post-anchoring specimen is shown in Figure 2d.

Pull-out testing was conducted using a dedicated anchorage testing system, equipped with synchronized load and displacement sensors. Force–displacement data were continuously recorded during loading to comprehensively evaluate anchoring performance under various conditions [32].

### 2.2. Test Conditions and Experimental Program

The parameters for this study were selected to represent a range of realistic scenarios encountered in underground engineering and to allow for a systematic investigation of their influence. The anchorage lengths of 400 mm, 600 mm, and 800 mm cover the typical range for short-to-medium length resin bolts used in roadway support, a common practice in coal mine engineering [33]. Similarly, the borehole water contents of 10%, 20%, and 30% (by volume) were chosen to simulate conditions ranging from damp to severely water-logged, which are frequently encountered in deep, fractured rock masses. Using these graduated values enables a systematic analysis of each parameter’s effect on anchoring performance.

**Figure 3:**
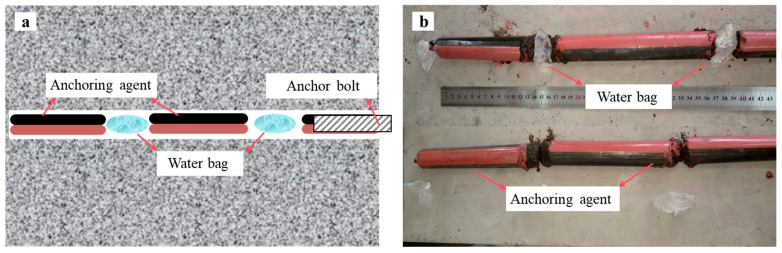
Staggered arrangement of resin and water bags: (**a**) Design scheme. (**b**) Actual layout.

To investigate the influence of water content and anchorage length on anchoring performance, tests were conducted under combinations of four water content levels (0%, 10%, 20%, and 30% of the borehole volume) and three anchorage lengths (400 mm, 600 mm, and 800 mm), as detailed in Table 1. Water content was controlled by uniformly placing customized water bags within the boreholes. By adjusting the number and volume of individual water bags, precise control over total moisture content was achieved. These bags were distributed along the bolt axis to ensure water–resin–steel interaction occurred consistently along the depth of the borehole (Figure 3).

Pull-out tests were conducted using the specialized anchorage testing system to capture real-time variations in anchorage force and displacement, allowing analysis of performance evolution under different conditions. In parallel, specimens of the same MSZ2360 resin incorporating water contents of 29 mL, 59 mL, and 98 mL were prepared for uniaxial compression testing, allowing for a direct evaluation of the impact of moisture on the commercial anchoring agent’s strength development.

### 2.3. Test Results and Analysis

#### 2.3.1. Uniaxial Compressive Strength of Resin with Water Content

**Figure 4:**
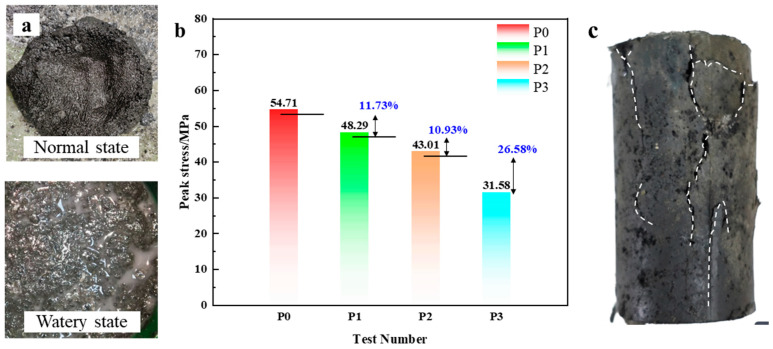
Uniaxial compressive test results of resin anchoring agents with different water contents: (**a**) Water absorption state. (**b**) Variation in peak compressive strength. (**c**) Typical failure morphology of specimen P3.

Figure 4 shows the uniaxial compressive strength results for the MSZ2360 resin samples with varying water contents. Specimens P0 to P3 correspond to water additions of 0 mL, 29 mL, 59 mL, and 98 mL, respectively. Water was observed to precipitate on the surface of the resin after mixing, as illustrated in Figure 4a. The peak compressive strength of the control specimen (P0) cured under natural conditions was 54.71 MPa, with a corresponding peak strain of 1.68%. As water content increased, compressive strength decreased significantly: P1 (29 mL) reached 48.29 MPa, P2 (59 mL) 43.01 MPa, and P3 (98 mL) 31.58 MPa—representing strength reductions of 11.73%, 21.39%, and 42.28%, respectively, relative to the dry specimen (Figure 4b). Peak strains increased correspondingly to 2.10%, 2.37%, and 2.62%.

These results indicate that water has a pronounced weakening effect on the mechanical properties of the anchoring resin. As water content increases, the resin exhibits softening behavior with greater peak strain and more easily propagating cracks, as observed in Figure 4c. Given that resin strength is a critical factor affecting overall anchorage stability, its performance deterioration under moist conditions results in significantly reduced load-bearing capacity and increases the likelihood of premature failure of the support system. Thus, in groundwater-rich or poorly drained boreholes, the interaction between water and resin must be carefully managed.

It is important to contextualize these findings. The peak compressive strength of 54.71 MPa for the control specimen (P0) under dry conditions aligns well with the typical performance range of 50–65 MPa specified by manufacturers for this class of commercial medium-speed polyester resin anchoring agents. This confirms that our baseline results are representative of standard materials used in the field. Therefore, the key finding of this section is not the material’s absolute strength, but the quantification of its significant performance deterioration—with a strength reduction of up to 42.28%—when exposed to water. This provides a crucial benchmark for the material improvement investigations presented in the subsequent sections.

#### 2.3.2. Pull-Out Performance Under Water-Bearing Conditions

**Figure 5:**
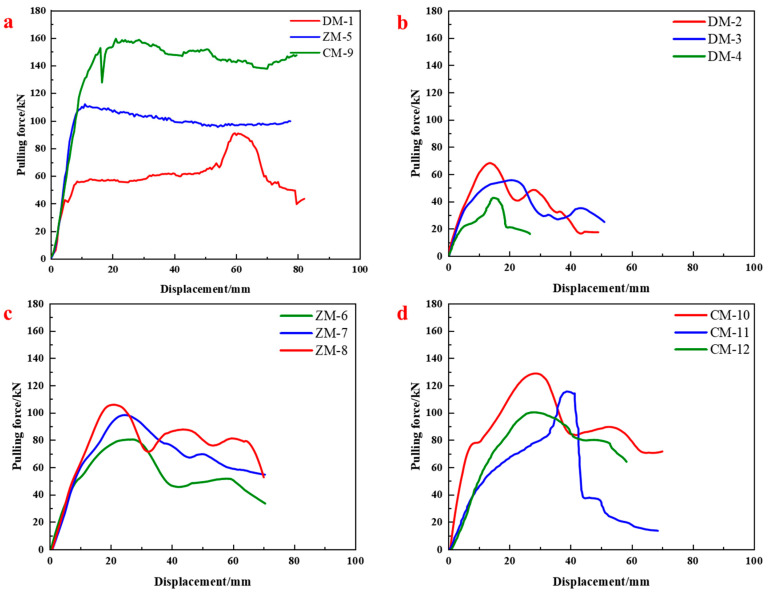
Pull-out load–displacement curves of resin anchorage under different water contents: (**a**) Dry borehole; (**b**) 10% water content; (**c**) 20% water content; (**d**) 30% water content.

The load–displacement curves from the pull-out tests under various conditions are presented in Figure 5. Figure 5a shows the influence of anchorage length under dry conditions. As the anchorage length increased from 400 mm to 600 mm, the peak pull-out force increased by 21.92%. Further increasing the length to 800 mm resulted in a 44.72% gain over the 600 mm case. This confirms that longer anchorage lengths improve the bond between resin, bolt, and surrounding rock, thereby enhancing system capacity. However, it was also observed that excessively long anchorage lengths may impair resin mixing uniformity—especially under rapid setting conditions—leading to disturbed initial bonding near the insertion end and a decline in effective anchorage performance beyond a suitable range.

Figure 5b–d depict the pull-out behavior under water contents of 10%, 20%, and 30%, respectively. Moisture significantly reduced the peak pull-out force of the anchorage system, a finding consistent with the observed reduction in the resin’s intrinsic compressive strength. At 30% water content, strength reduction exceeded 40% for end-anchored specimens. While increased anchorage length offered some compensatory effect under the same water content, this benefit diminished progressively with further length increases.

**Figure 6:**
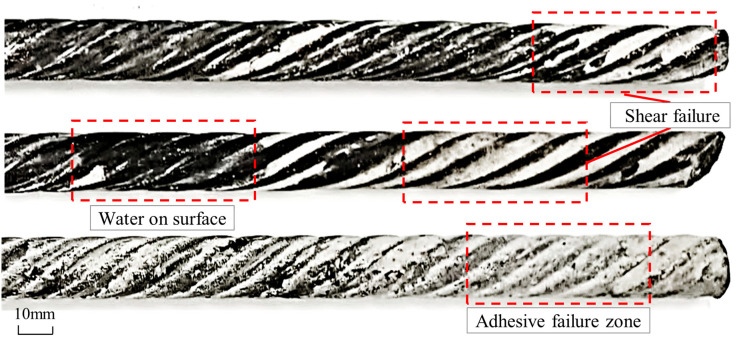
Schematic illustration of anchorage pull-out failure.

Failure mode analysis under wet conditions (using 400 mm specimens, Figure 6) revealed that all specimens failed via resin–bolt interface debonding. The resin was sheared and stripped from the bolt threads, and higher moisture levels resulted in more uncured resin residue within the helical grooves. The failed resin exhibited a soft, plastic state, indicating incomplete curing even after 24 h, suggesting that water not only delays but may inhibit the resin setting process. The bolt surfaces were visibly moist, and competition for borehole volume between resin and water resulted in void formation and reduced density, compromising the cooperative load-bearing function of the anchorage system.

## 3. Experimental Study on the Formulation Improvement of Resin Anchoring Agent

To address the widespread issues of high water content and acid–alkaline corrosion encountered during deep coal mine roadway support, conventional resin anchoring agents often exhibit inadequate water resistance and chemical durability, limiting their stable application under complex environmental conditions. To improve environmental adaptability and structural stability, this study introduces an improved resin formulation and systematically evaluates its water resistance and mechanical performance. The objective is to develop a high-performance resin anchoring system suitable for complex geological conditions, thereby ensuring the safe and reliable operation of anchorage systems in deep underground environments.

### 3.1. Strategy for Formulation Improvement and Material Selection

The formulation improvement of the resin anchoring agent was approached from two key aspects: (1) Enhancing the filler-to-resin mass ratio and adjusting the particle size distribution of the fillers to improve the compactness and mechanical performance of the anchoring agent; (2) Incorporating hydroxypropyl acrylate resin and a super absorbent polymer (SAP) to improve water resistance.

**Figure 7:**
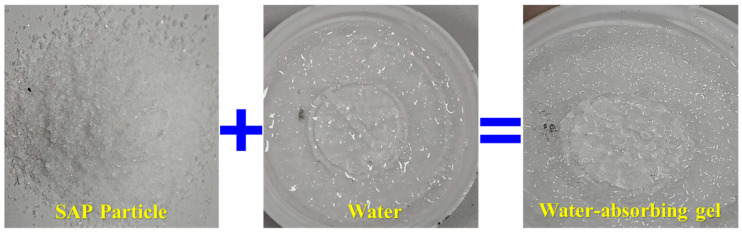
Water absorption and gelation process of SAP.

As a functional additive, SAP exhibits instantaneous water absorption, capable of absorbing several hundred times its own volume in water. It can effectively inhibit water diffusion within the resin matrix, thereby reducing interference with the curing process and improving the material’s stability and long-term performance (Figure 7).

The resin matrix was formulated using a blend of unsaturated polyester resin (PET) and hydroxypropyl acrylate resin. The filler consisted of a mixture of coarse (20–40 mesh) and fine (160–200 mesh) stone powders. To further enhance dispersion and reactivity, silane coupling agent (KH560), dispersants, benzoyl peroxide (BPO), and accelerators such as dimethylaniline (DMA) and dimethyl-p-toluidine (DMT) were added.

### 3.2. Material Formulation and Optimization

Given the high proportion of fillers in resin anchoring agents, their particle size composition and filler-to-resin ratio significantly affect both rheological behavior and mechanical properties. The study first investigated a suitable filler-to-resin mass ratio (groups PT-1 to PT-4), starting with an initial ratio of 1:5. Based on the selected ratio, the influence of coarse-to-fine filler ratios on matrix stability was further analyzed to determine the most suitable filler combination.

Subsequently, hydroxypropyl acrylate resin was introduced to enhance resistance to water and chemical erosion. Widely used in waterproof coatings, this resin provides excellent media resistance. Due to its high fluidity, which negatively impacts structural retention when used alone, it was co-blended with PET in the formulation. This allowed the formation of an interpenetrating polymer network with fillers, improving both adaptability and stability under water-bearing conditions.

Additionally, SAP was incorporated as a variable component to form a micro-scale water barrier within the resin, thereby retarding water diffusion, mitigating curing interference, and improving structural compactness and mechanical strength. The experimental variable in this section was the SAP content, with a focus on its effectiveness in enhancing water resistance.

The dosage of curing agent, coupling agent, and accelerators was controlled at 6%, 1%, and 1% of the total paste mass, respectively. The detailed formulation for each group is presented in Table 2.

### 3.3. Characterization Methods

To evaluate the performance of the different formulations, both the mechanical properties of the cured solid and the rheological properties of the fresh paste were characterized.

For each formulation, three cylindrical specimens (50 mm in diameter and 100 mm in height) were cast. After curing for 7 days under standard laboratory conditions (20 ± 2 °C and >95% relative humidity), the specimens were tested for uniaxial compressive strength using a universal testing machine. The test was conducted in accordance with ASTM D695 standards at a constant loading rate of 2 mm/min. The peak stress was recorded as the compressive strength [34].

The flowability and workability (often termed consistency) of the freshly prepared resin paste were measured. The test was performed using a flow spread method, in accordance with the Chinese national standard GB/T 2419-2005 [35,36]. A standardized conical mold was filled with the fresh resin paste and placed at the center of a horizontal glass plate. The mold was then lifted vertically, allowing the paste to spread under its own weight for 180 s. The final spread diameter was measured in two perpendicular directions, and the average value was recorded in millimeters (mm) as the flow spread. A larger flow spread diameter indicates lower viscosity and better flowability.

### 3.4. Material Performance Results and Analysis

#### 3.4.1. Effect of Filler Content on Compressive Strength

**Figure 8:**
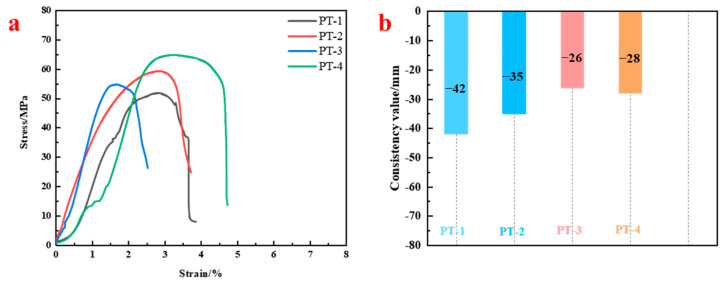
Mechanical performance and flow spread of resin anchoring agents with different filler contents: (**a**) Stress–strain curves. (**b**) Flow spread test results.

Figure 8 shows the relationship between uniaxial compressive strength and flow spread of resin anchoring agents under different filler contents. Peak compressive strengths for groups PT-1 to PT-4 were 51.80 MPa, 59.19 MPa, 54.68 MPa, and 64.71 MPa, respectively (Figure 8a). The results demonstrate that increasing the filler-to-resin mass ratio generally enhances compressive strength. Specifically, when the ratio increased from 5:1 to 5.6:1, strength improved by nearly 25%, indicating a strong filler reinforcement effect.

However, further increases in filler content significantly reduced material flowability, as evidenced by smaller flow spread diameters, indicating reduced workability, in the test (Figure 8b). In practical applications, reduced fluidity leads to increased bolt insertion resistance, which may affect construction efficiency and anchoring quality. Therefore, considering both mechanical performance and workability, the PT-2 formulation with a 5.2:1 mass ratio is recommended as the most suitable mix.

#### 3.4.2. Effect of Coarse-to-Fine Filler Ratio on Strength

**Figure 9:**
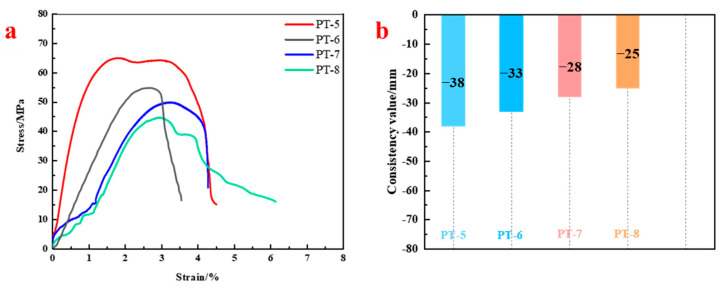
Mechanical performance and flow spread of resin anchoring agents with different coarse-to-fine filler ratios: (**a**) Stress–strain curves; (**b**) Flow spread test results.

Figure 9 illustrates the influence of different coarse-to-fine filler ratios on compressive strength and flow spread. Peak compressive strengths of PT-5, PT-6, PT-7, and PT-8 were 64.90 MPa, 54.66 MPa, 49.74 MPa, and 44.60 MPa, respectively.

These results are governed by the principles of particle packing theory, a fundamental concept in the design of high-performance composite materials. The coarse filler particles form a load-bearing skeletal structure, where compressive stress is efficiently transferred through inter-particle locking, enhancing the overall matrix stability. The fine filler particles then occupy the interstitial voids between the coarse particles, which increases the packing density of the solid phase and minimizes the volume of the relatively weaker resin binder required.

This principle is directly analogous to the design of well-graded aggregates in systems like high-performance concrete and polymer mortars, where a well-graded particle size distribution is critical for maximizing compressive strength and minimizing binder demand [37,38]. A deviation from the effective ratio disrupts this synergy. An excessive coarse content leads to insufficient fines to fill the voids, resulting in higher porosity and poorly supported grains. Conversely, an excess of fine particles dramatically increases the total specific surface area of the filler, leading to an insufficient resin film for effective bonding and a sharp decrease in workability (flowability) due to increased inter-particle friction [39].

Therefore, the PT-6 group (coarse-to-fine mass ratio of 1.2:0.8) represents the most effective combination in our system, achieving a balance between high packing density for strength and sufficient resin content for workability and bonding.

#### 3.4.3. Effect of Resin Composition on Strength

**Figure 10:**
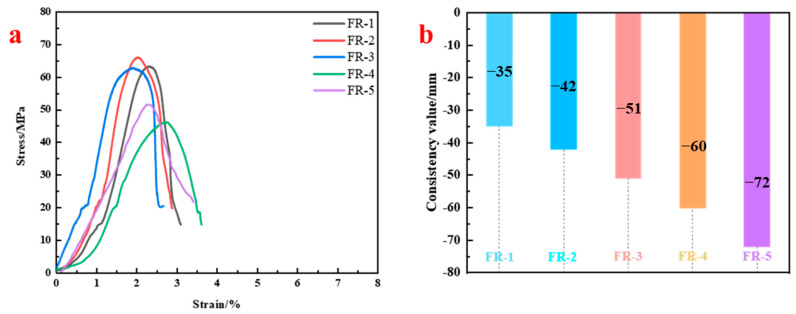
Mechanical performance and flow spread of resin anchoring agents with different resin compositions: (**a**) Stress–strain curves; (**b**) Flow spread test results.

Based on the selected filler combination, various co-blending ratios of hydroxypropyl acrylate and PET resins were evaluated to assess their effect on anchoring performance. Figure 10 presents compressive strength and flow spread for each formulation. Groups FR-1 to FR-3 showed uniaxial compressive strengths of 63.13 MPa, 65.96 MPa, and 62.67 MPa, all exceeding the reference PT-6 group. However, when the proportion of hydroxypropyl acrylate resin dominated (FR-4 and FR-5), strength declined significantly to 46.03 MPa and 51.47 MPa, approximately 20 MPa lower than FR-2. Meanwhile, the flow spread increased significantly with higher acrylate content. For instance, FR-5 showed a flow spread diameter of 72 mm, nearly twice that of FR-1, indicating enhanced fluidity and extended setting time.

These results suggest that although hydroxypropyl acrylate resin improves flowability and water resistance, its bonding ability with inorganic fillers and post-curing structural stability is inferior to PET. Thus, a PET-dominated co-blend (resin ratio of 0.7:0.3 in FR-1) is recommended as the most effective formulation.

#### 3.4.4. Water Resistance Evaluation of the Improved Formulation

Based on the above material development, FR-1 was selected as the recommended formulation. To further evaluate its mechanical stability under wet conditions, comparative tests were conducted by incorporating SAP at mass fractions of 0.5%, 1.0%, and 2.0%. The corresponding specimens were labeled Fa, Fb, and Fc. The test matrix is shown in Table 3.

**Figure 11:**
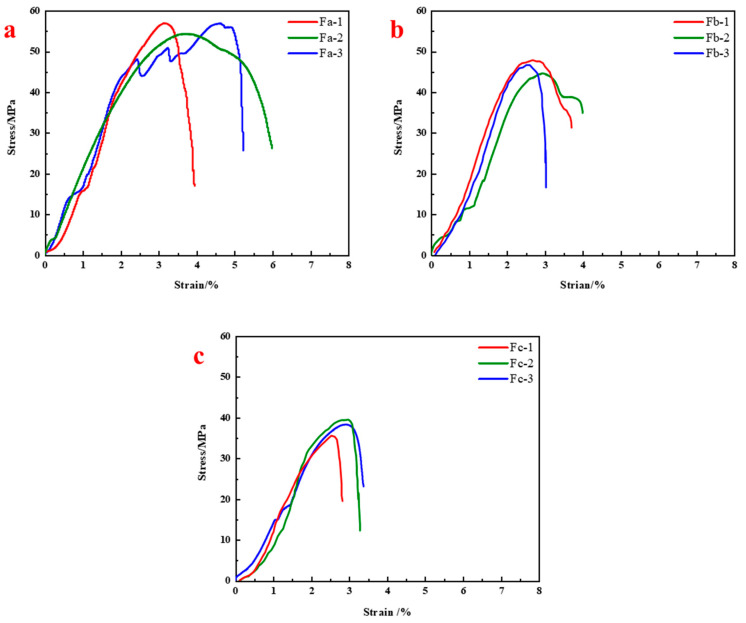
Water-resistant mechanical performance of resin anchoring agents with improved formulation: (**a**) SAP content = 0.5%; (**b**) SAP content = 1.0%; (**c**) SAP content = 2%.

Test results (Figure 11) show that compressive strengths of Fa (0.5% SAP) were 56.86 MPa, 56.92 MPa, and 54.34 MPa, corresponding to strength reductions of 9.93%, 9.83%, and 13.92% relative to FR-1. For Fb (1.0% SAP), the strengths were 46.66 MPa, 47.85 MPa, and 44.58 MPa, with reductions of 26.08%, 24.20%, and 29.38%. For Fc (2.0% SAP), values further dropped to 38.30 MPa, 39.54 MPa, and 35.58 MPa, representing losses of 39.33%, 37.36%, and 43.64%, respectively. These results confirm that the improved formulation demonstrates significantly higher strength retention under water interference compared to conventional resins, indicating superior water resistance and structural integrity.

Further analysis suggests that moderate SAP addition (0.5–1.0%) effectively inhibits water-induced structural degradation and improves curing quality and compressive strength in moist conditions. However, at 2.0% SAP content, strength declined significantly despite still outperforming unimproved formulations. Visual inspection revealed local cracks on Fc samples, likely due to: (1) SAP swelling stress concentration, (2) poor dispersibility and agglomeration in high-viscosity resin, and (3) excessive SAP volume leading to porosity and shrinkage-induced cracks during curing, ultimately compromising structural integrity.

## 4. Experimental Study on Acid–Alkali Corrosion Performance at the Resin–Rock Interface

In deep coal mine roadways, support systems are often exposed to chemically aggressive groundwater over long periods. Chemical corrosion at the resin–rock interface can significantly reduce bonding strength, thereby compromising the overall safety and stability of the support system [15]. To investigate the effects of acidic and alkaline environments on the mechanical and microstructural behavior of the resin–rock interface, this study conducted comparative shear and SEM analyses under three representative pH conditions (acidic, neutral, and alkaline). These experiments aim to provide a theoretical basis for evaluating material adaptability and guiding formulation improvement in chemically aggressive underground environments.

### 4.1. Experimental Materials

The rock material used to simulate surrounding rock was weakly cemented silty sandstone with an average uniaxial compressive strength of approximately 25 MPa, representing the soft rock commonly encountered in underground coal mines [25]. Two types of resin anchoring agents were selected: a conventional commercial formulation and an improved formulation developed in this study. To evaluate the bonding performance at the resin–rock interface, customized stainless-steel ring shear molds were used. Each type of resin was evenly applied to the end surface of the sandstone to fabricate standardized interface specimens.

To simulate typical groundwater chemistry, three types of aqueous solutions with controlled pH values were prepared: acidic (pH = 5, using 0.1 mol/L HCl), neutral (pH = 7, using distilled water), and alkaline (pH = 9, using 0.1 mol/L NaOH). In addition, five representative ions commonly found in aquifers of western China—Na^+^, K^+^, Mg^2+^, Cl^−^, and SO_4_^2−^—were considered due to their potential effects on interface degradation [26]. For safety and reproducibility, actual groundwater was not used; instead, representative chemical environments were synthesized in the laboratory.

### 4.2. Testing Method

**Figure 12:**
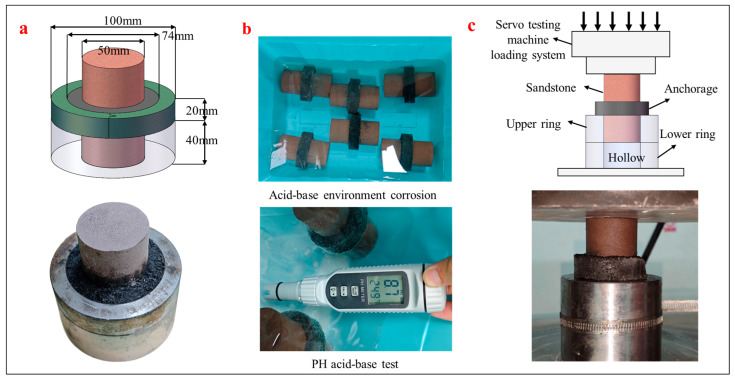
Schematic diagram of interfacial corrosion testing for resin anchoring agents: (**a**) Structure and dimensions of annular shear specimen; (**b**) Immersion process in different pH solutions; (**c**) Principle of annular shear loading.

To evaluate the bonding strength between the resin anchoring agents and the rock substrate under chemical attack, specimens were prepared using stainless-steel annular shear molds to ensure uniform and reliable bonding. The mold dimensions are shown in Figure 12a. The tested anchoring agents included a commercial MSZ2360 formulation and the newly improved resin formulation.

The specimens were immersed in the prepared solutions for 24 h, 48 h, and 72 h. These specific time points were deliberately chosen to capture the critical early stage of chemical attack, during which the most rapid chemical reactions at the resin–rock interface are expected to occur. As presented later in Section 4.3.1, periodic pH monitoring confirmed that the chemical system approached equilibrium within this 72 h period, thereby validating this timeframe as sufficient for assessing the initial performance deterioration. A schematic of the immersion process is shown in Figure 12b.

After the designated immersion period, annular shear tests were performed to evaluate interfacial shear strength and failure displacement (Figure 12c). Additionally, scanning electron microscopy (SEM) was used to observe the microstructural changes in the resin and interface after exposure, providing insights into the corrosion mechanisms.

### 4.3. Results and Discussion

#### 4.3.1. pH Evolution During Immersion

**Figure 13:**
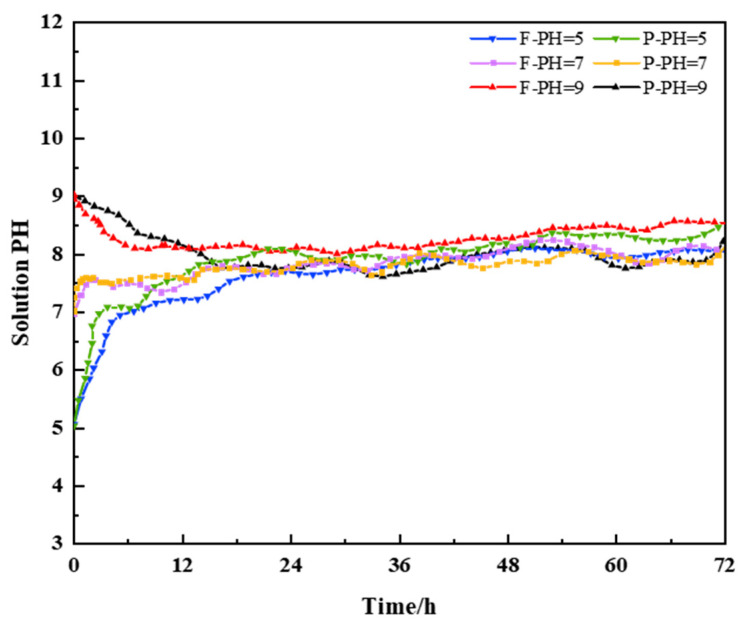
pH variation over time for resin–rock composites immersed in acidic and alkaline solutions.

During immersion, chemical reactions occurred between the anchoring agent and the solution, such as mineral dissolution, delamination, and matrix degradation. These processes increased internal porosity and compromised mechanical integrity. The pH variation trends of the acidic and alkaline solutions for both conventional and improved resin–sandstone composites are shown in Figure 13, where F denotes the improved resin and P denotes the conventional resin; s and j represent acidic and alkaline environments, respectively.

As shown, all pH values exhibited an initial rapid fluctuation followed by gradual stabilization. This indicates that the primary chemical reactions occurred in the early stages of immersion, and the system approached equilibrium over time.

#### 4.3.2. Interfacial Shear Behavior Under Initial Conditions

**Figure 14:**
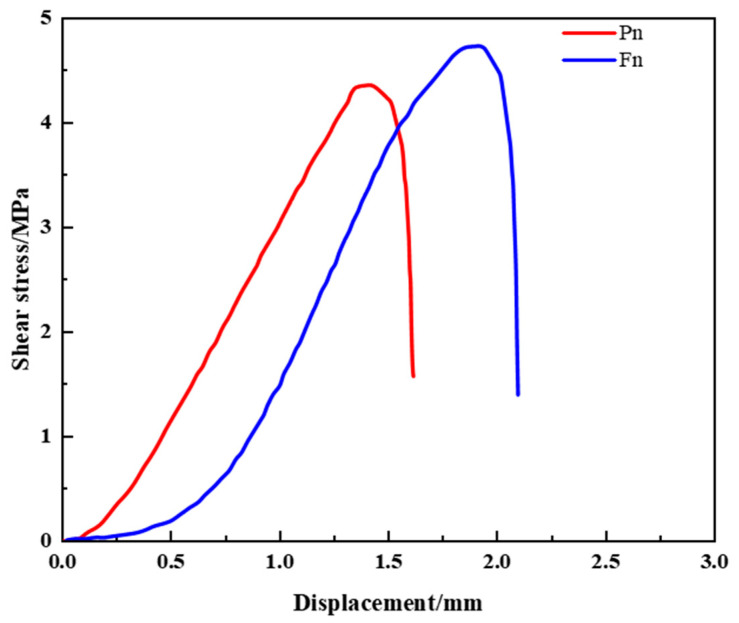
Shear–displacement curves of two types of resin anchoring agents under non-corrosive conditions.

The shear–displacement curves for the untreated specimens (i.e., before chemical exposure) are presented in Figure 14. For the conventional resin (Pn), the peak shear stress was 4.37 MPa with a failure displacement of 1.38 mm. In contrast, the improved resin (Fn) reached a peak shear stress of 4.77 MPa and failure displacement of 1.89 mm, representing increases of approximately 9% and 0.51 mm, respectively.

All curves exhibited three typical stages: an initial linear phase dominated by adhesive bonding at the interface, a transition phase characterized by reduced shear growth and increased displacement due to progressive failure and frictional engagement, and a final failure phase where bonding failed completely and interfacial slip occurred.

These results confirm that the improved resin provides improved bonding strength and ductility, enhancing both interface cohesion and load-carrying capacity.

#### 4.3.3. Interfacial Shear Behavior After Chemical Exposure

**Figure 15:**
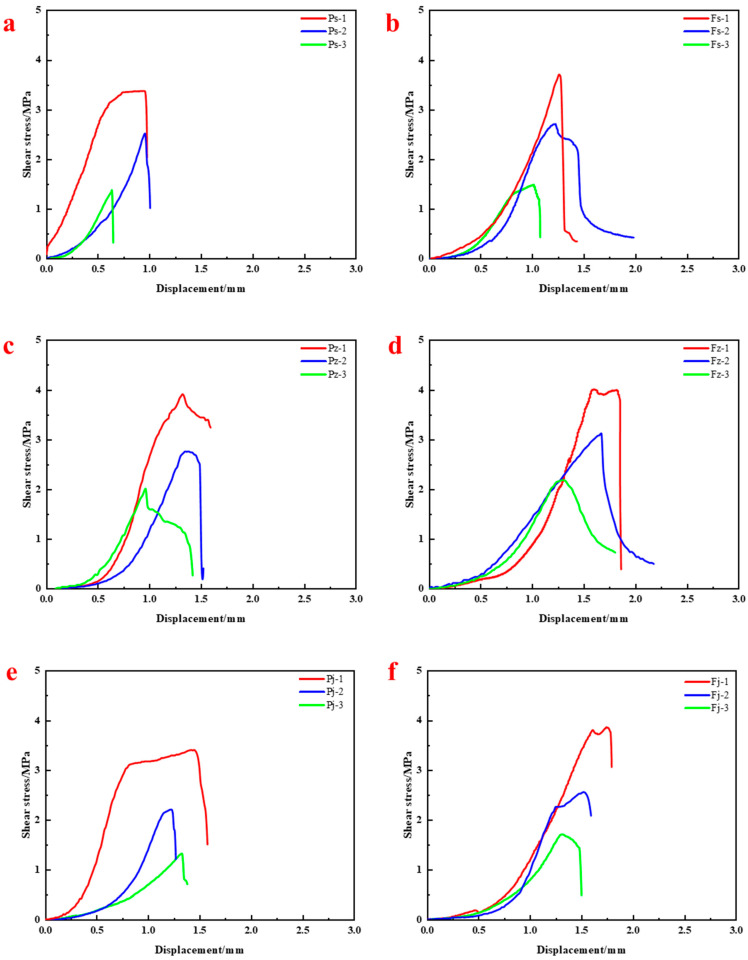
Shear–displacement responses of resin–rock interfaces under acidic and alkaline environments: (**a**) Conventional resin in acidic environment; (**b**) Improved resin in acidic environment; (**c**) Conventional resin in neutral environment; (**d**) Improved resin in neutral environment; (**e**) Conventional resin in alkaline environment; (**f**) Improved resin in alkaline environment.

Figure 15 shows the evolution of shear–displacement curves after immersion in acidic (pH = 5), neutral (pH = 7), and alkaline (pH = 9) environments for 24 h, 48 h, and 72 h.

The conventional resin exhibited severe degradation in all environments, with both strength and ductility declining as immersion time increased. Under acidic conditions, shear strength dropped to 3.38 MPa, 2.50 MPa, and 1.33 MPa, and failure displacement declined to 0.95 mm, 0.94 mm, and 0.63 mm, respectively (Figure 15a), indicating a transition to brittle failure. Under neutral conditions (Figure 15c), strengths were 3.91 MPa, 2.76 MPa, and 2.17 MPa with minor displacement variation. Under alkaline conditions (Figure 15e), shear strengths were 3.41 MPa, 2.21 MPa, and 1.32 MPa, and displacements remained relatively stable.

In contrast, the improved resin showed better retention of interfacial shear strength and ductility. In acidic solutions, shear strengths were 3.69 MPa, 2.72 MPa, and 1.47 MPa, with corresponding displacements of 1.25 mm, 1.22 mm, and 1.01 mm. In neutral conditions, strengths were 4.01 MPa, 3.11 MPa, and 2.18 MPa with displacements of 1.83 mm, 1.66 mm, and 1.30 mm. In alkaline environments, strengths were 3.86 MPa, 2.56 MPa, and 1.71 MPa, with displacements of 1.75 mm, 1.52 mm, and 1.47 mm.

These findings indicate that the improved resin significantly enhances interfacial load-bearing capacity and deformation compatibility, making it more suitable for long-term service under chemically aggressive conditions.

#### 4.3.4. Failure Mode Analysis

**Figure 16:**
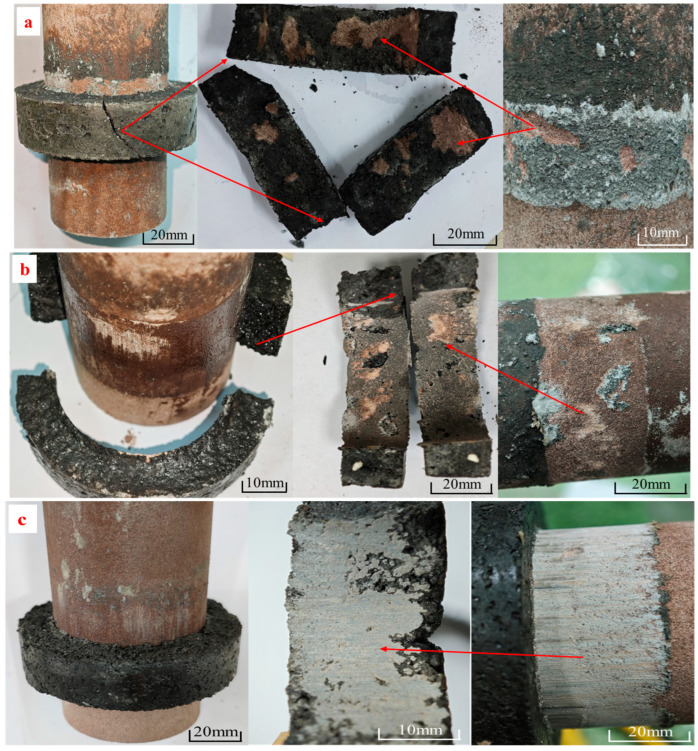
Typical interfacial failure modes under different pH environments: (**a**) Type I: Multi-segment radial delamination (acidic/alkaline); (**b**) Type II: Bipartite delamination (neutral); (**c**) Type III: Complete interfacial slip (due to preparation defects).

After immersion, three typical interfacial failure modes were observed (Figure 16):

Type I: Multi-fragment radial delamination, mainly occurred under acidic (pH ≈ 5) and alkaline (pH ≈ 9) conditions. Corrosive media attacked the resin–mineral interface, causing bonding degradation. When critical shear stress was reached, the resin ring fractured into three or more curved segments. Though resin residues were observed, average shear strength dropped by 45–60%. Longer immersion (7 to 28 days) did not alter the macroscopic failure type but increased the depth of chemical erosion.

Type II: Bipartite delamination, mostly observed under neutral conditions (pH ≈ 7). Localized micro-cracks allowed solution ingress, forming pitting and weakening the interface. Failure resulted in two arc-shaped segments with continuous resin remnants. Shear strength reduction remained below 25%, indicating better load transfer and ductility than Type I.

Type III: Complete interfacial slip, accounted for <5% of cases and was not strongly correlated with pH. The resin ring detached entirely with no bonding residue. This failure mode was attributed to poor mixing or surface contamination during specimen preparation rather than chemical corrosion.

These results underscore the importance of pH-driven corrosion on resin interface failure modes. While neutral environments are relatively stable, Type III failure highlights the necessity of proper grouting and interface cleaning in engineering practice.

#### 4.3.5. Microstructural Analysis

**Figure 17:**
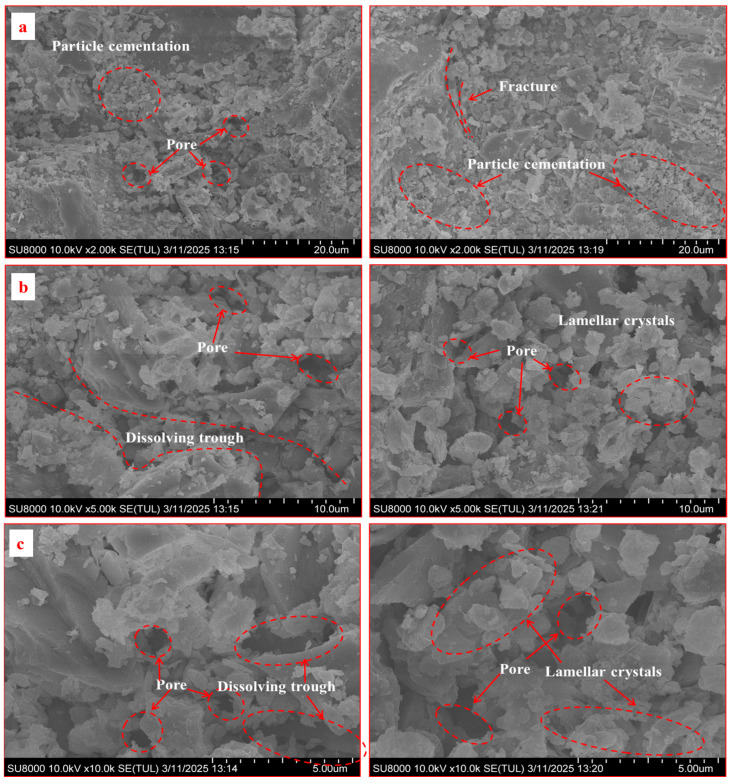
SEM comparison of two types of resin anchoring agents under acidic conditions (pH = 5) (**Left**: conventional resin; **Right**: improved resin): (**a**) ×2000; (**b**) ×5000; (**c**) ×100,000 magnification.

**Figure 18:**
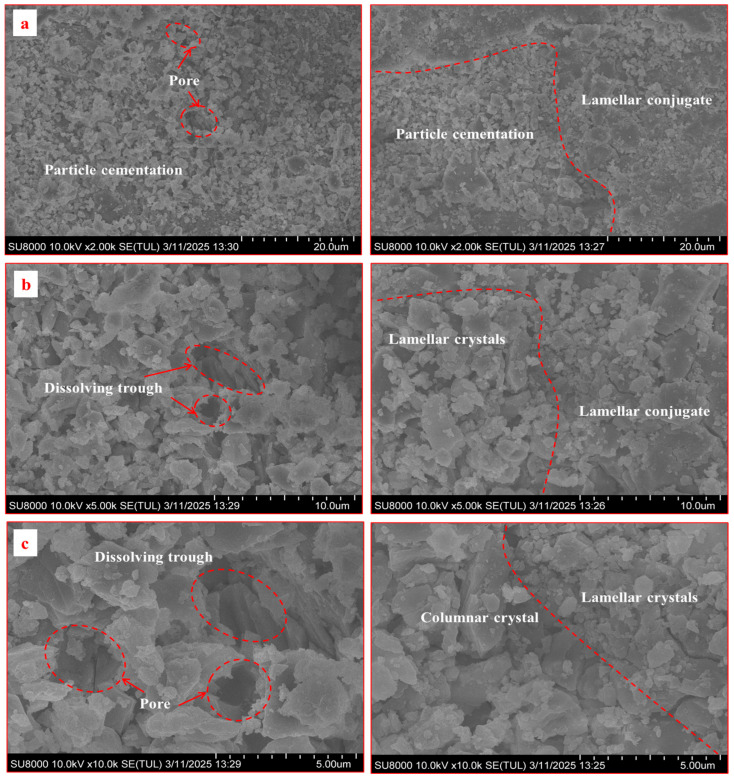
SEM comparison of two types of resin anchoring agents under alkaline conditions (pH = 9) (**Left**: conventional resin; **Right**: improved resin): (**a**) ×2000; (**b**) ×5000; (**c**) ×100,000 magnification.

SEM observations at magnifications of 2 × 10^3^, 5 × 10^3^, and 1 × 10^5^ were conducted on resin specimens after chemical immersion (Figure 17 and Figure 18).

In acidic conditions (Figure 17), the conventional resin exhibited widespread irregular pores, flocculent gel residues, and pronounced laminar delamination, reflecting a combined “debonding–dissolution” failure. In contrast, the improved resin—containing corrosion-resistant polymers and SAP—showed compact grain contacts, tight lamellar arrangements, minor etching pits, and better structural integrity, indicating superior microstructural stability.

Under alkaline conditions (Figure 18), fewer pores and cracks were observed. Local crystal detachment occurred due to mild hydrolysis and filler decalcification. The improved resin retained a well-defined crystal framework with dense laminar coupling structures, confirming its superior alkali resistance.

Overall, both acidic and alkaline media induce hydrolysis and interface decalcification, initiating irreversible microstructural degradation. Microcrack propagation and pore growth underlie the macroscopic decline in shear and tensile strength. The improved formulation, by incorporating acid–alkali-resistant resins and SAP, enhanced both chemical stability and microcrack suppression through swelling and gap-filling, delaying failure evolution.

These findings demonstrate that microstructural degradation is the root cause of mechanical deterioration under chemical corrosion. The proposed formulation improvement strategy significantly improves resistance and durability, offering a robust basis for selecting anchoring materials in aggressive underground environments.

## 5. Discussion

This study provides a systematic evaluation of resin anchor performance in hydrochemical environments and presents an improved formulation designed to mitigate the observed deterioration. The results offer several key points for discussion regarding the mechanisms of performance loss and the effectiveness of the material improvement strategy.

### 5.1. Mechanism of Performance Deterioration in Conventional Resin

Our findings clearly demonstrate that the presence of even moderate amounts of water (10–30% by volume) leads to a drastic reduction in the mechanical performance of conventional polyester resin anchors, with compressive strength dropping by over 40%. This aligns with the findings of Kang et al. [9] and Wang et al. [10], who also reported significant reductions in anchorage capacity in water-bearing conditions. Our work expands on this by showing a clear shift in the failure mode from cohesive failure within the resin body to interfacial debonding. This suggests that water’s primary detrimental effect is not just on the bulk material strength but, more critically, on the integrity of the resin–bolt and resin–rock interfaces. The observed presence of uncured, soft resin residue (Figure 6) confirms that water physically interferes with the polymerization process, likely by preventing complete cross-linking and creating a weak, plasticized boundary layer at the interface, as also suggested by Wang et al. [11].

Furthermore, the severe drop in interfacial shear strength (over 70%) in acidic environments (pH = 5) corroborates the mechanism of ester bond hydrolysis described by previous researchers [15,16]. Our results quantitatively demonstrate that this chemical attack is rapid, with most of the damage occurring within 72 h. This finding has significant implications for the long-term durability of rock bolts in mines with acidic drainage, suggesting that the service life of conventional resin anchors may be significantly overestimated if such chemical effects are not considered.

### 5.2. Efficacy and Mechanisms of the Improved Formulation

The central contribution of this work is the development of an improved resin formulation. The strategy of combining a co-blended polymer matrix (PET and hydroxypropyl acrylate) with a functional additive (SAP) proved highly effective. The superior performance of this new material can be attributed to a synergistic, multi-faceted mechanism.

First, the incorporation of SAP acts as an internal water sink. Instead of allowing free water to interfere with polymerization, the SAP rapidly sequesters it into a stable hydrogel, as illustrated in Figure 7. This protects the curing reaction and allows for the formation of a more complete and robust polymer network. This mechanism is analogous to the use of SAP to mitigate internal curing stresses and improve hydration in high-performance concrete [40]. Our results, showing strength retention of over 85% in saturated conditions, provide strong evidence for the success of this internal water management strategy.

Second, the partial replacement of PET with hydroxypropyl acrylate (HPA) enhances the chemical stability of the matrix. HPA is known for its superior resistance to hydrolysis compared to standard unsaturated polyesters. This likely accounts for the improved retention of interfacial shear strength in acidic and alkaline environments. The SEM images (Figure 17 and Figure 18) support this conclusion, revealing a much denser and less fractured microstructure for the improved resin after chemical exposure. While the conventional resin showed widespread dissolution and delamination, the improved resin maintained its inter-granular bonding and structural integrity.

### 5.3. Practical Implications and Study Limitations

The findings of this study have significant practical implications for ground support in challenging geological conditions. The proposed formulation offers a tangible solution to a well-known engineering problem. The use of a balanced filler gradation, as informed by particle packing theory, and the incorporation of functional additives like SAP represent a material design strategy that could be widely adopted to enhance the safety and reliability of underground constructions.

However, some limitations should be acknowledged. This study was conducted under laboratory conditions using simulated rock (concrete) and synthesized chemical solutions. The complex stress states, temperature variations, and synergistic effects of multiple ions in real groundwater were not fully replicated. Furthermore, the tests were limited to a 72 h period to capture the acute phase of deterioration. The long-term performance over months or years, where creep and fatigue mechanisms become more prominent, requires further investigation. Therefore, while this study provides a robust proof-of-concept, long-term field validation is a critical next step before widespread implementation.

## 6. Conclusions

This study makes a significant contribution by developing and validating a high-performance, water- and chemical-resistant resin anchoring agent, providing a robust material solution for the known problem of support degradation in challenging underground environments. The primary novel findings and their implications are:

1. The key scientific contribution is a new material design strategy. We have demonstrated that the synergistic combination of a modified polymer matrix (using hydroxypropyl acrylate) and an internal water-mitigating agent (Super Absorbent Polymer) can successfully overcome the well-documented performance limitations of conventional polyester resins in hydrochemical environments.
2. This work provides new mechanistic insights. We established a clear link between the improved macroscopic performance (i.e., strength retention and interfacial durability) and the material’s microstructure. The evidence shows that the improved formulation maintains its structural integrity by suppressing the initiation and propagation of microcracks at the resin–rock interface, which is a critical failure mechanism.
3. The findings have significant practical implications. This study provides not just a new material but a validated formulation approach that can be used to enhance the safety and long-term reliability of ground support systems in deep and geologically complex mines.

Future research should build upon these findings, focusing on long-term field validation. Furthermore, developing detailed numerical models (e.g., using the Finite Element Method, FEM) to simulate the quantitative patterns of material deterioration will be crucial for creating a more comprehensive understanding and contributing to robust design standards.

## Figures and Tables

**Table 1:** Test matrix for pull-out experiments under water-bearing conditions.

Specimen ID	Anchorage Length (mm)	Borehole Water Content (vol%)
DM-1	400	0
DM-2		10
DM-3		20
DM-4		30
ZM-5	600	0
ZM-6		10
ZM-7		20
ZM-8		30
CM-9	800	0
CM-10		10
CM-11		20
CM-12		30

**Table 2:** Experimental design for formulation improvement of resin anchoring agents.

Specimen ID	Unsaturated Polyester Resin (PET)	Hydroxypropyl Acrylate Resin	Coarse Filler	Fine Filler
PT-1	1.00		2.50	2.50
PT-2	1.00		2.60	2.60
PT-3	1.00		2.70	2.70
PT-4	1.00		2.80	2.80
PT-5	1.00		0.55 × a	0.45 × a
PT-6	1.00		0.60 × a	0.40 × a
PT-7	1.00		0.65 × a	0.35 × a
PT-8	1.00		0.70 × a	0.30 × a
FR-1	0.70	0.30	b	c
FR-2	0.65	0.35	b	c
FR-3	0.50	0.50	b	c
FR-4	0.30	0.70	b	c
FR-5	0.15	0.85	b	c

Note: “a” represents the total filler mass in PT-1 to PT-4; “b” and “c” refer to the coarse and fine filler masses in PT-5 to PT-8, respectively.

**Table 3:** Improved formulation design for water-resistant resin anchoring agents with varying SAP contents.

Specimen ID	Water Addition (mL)	SAP Content (%)
Fa-1	29	0.5
Fa-2		1.0
Fa-3		2.0
Fb-1	59	0.5
Fb-2		1.0
Fb-3		2.0
Fc-1	98	0.5
Fc-2		1.0
Fc-3		2.0

## Data Availability

The raw data supporting the conclusions of this article will be made available by the authors on request.
